# The Importance of NADPH Oxidases and Redox Signaling in Angiogenesis

**DOI:** 10.3390/antiox6020032

**Published:** 2017-05-13

**Authors:** Rodrigo Prieto-Bermejo, Angel Hernández-Hernández

**Affiliations:** Department of Biochemistry and Molecular Biology, University of Salamanca, Salamanca 37007, Spain; rodrix@usal.es

**Keywords:** ROS, NADPH oxidase (Nox), redox signaling, angiogenesis

## Abstract

Eukaryotic cells have to cope with the constant generation of reactive oxygen species (ROS). Although the excessive production of ROS might be deleterious for cell biology, there is a plethora of evidence showing that moderate levels of ROS are important for the control of cell signaling and gene expression. The family of the nicotinamide adenine dinucleotide phosphate oxidases (NADPH oxidases or Nox) has evolved to produce ROS in response to different signals; therefore, they fulfil a central role in the control of redox signaling. The role of NADPH oxidases in vascular physiology has been a field of intense study over the last two decades. In this review we will briefly analyze how ROS can regulate signaling and gene expression. We will address the implication of NADPH oxidases and redox signaling in angiogenesis, and finally, the therapeutic possibilities derived from this knowledge will be discussed.

## 1. The Importance of ROS in Cellular Signaling and Gene Expression

During evolution, the access to oxygen was a turning point for eukaryotic cells, because they were able to develop a much more efficient energetic metabolism compared to anaerobic conditions. However, aerobic metabolism brings with it the production of reactive oxygen species (ROS) by the partial reduction of oxygen, the most common superoxide (O^−^_2_), hydrogen peroxide (H_2_O_2_), and the hydroxyl radical (OH^−^) [1]. A high ROS production can be related to cell damage and disease [2], since these molecules are highly reactive and can oxidize all kinds of biomolecules [3]. Oxidative stress is detrimental to the cells and can be linked to aging [4] and the development of degenerative diseases, including cancer [5,6]. Nevertheless, it is also true that aerobic cells have adapted to a moderate production of ROS, to the point that ROS have become important in the regulation of signal transduction [7] and gene expression [8].

A long time ago, it was shown that H_2_O_2_ could mimic growth factor activity [9]. Moreover, it is also known that growth factors activate the production of H_2_O_2_ in cells [10]. ROS behave as important regulators of cellular signaling, and the redox signaling concept is currently broadly accepted [11]. ROS could be considered as bona fide second messengers because the cell has enzymatic systems that produce and eliminate them in a regulated manner. If we accept this notion, then a difficult question to answer is how ROS achieve specificity during cellular signaling. We are just starting to understand the molecular mechanisms through which ROS control signaling, and it would be very interesting if we could identify the thin line that separates the biological effects of ROS from oxidative damage.

The way in which ROS affect cellular signaling inevitably has to be the reversible oxidation of signaling molecules. Thus, to fully understand redox signaling, we need to identify these targets. Cysteine residues can easily be oxidized to sulfenic acid (–SOH), sulfinic acid (–SO_2_H), and sulfonic acid (–SO_3_H). Hence, proteins that have cysteine residues important for their function are very sensitive to oxidation [12].

Reversible phosphorylation is one of the most common mechanisms for regulating protein function, which is involved in the regulation of proliferation, differentiation, cell cycle control, and transcription regulation. The levels of protein phosphorylation in cells depend on the balance between kinases and phosphatases. There are more than 500 kinases encoded in the human genome [13], and around 150 phosphatases [14]. According to their specificity, they can be classified as serine/threonine (Ser/Thr) or tyrosine (Tyr) kinases or phosphatases. The number of Ser/Thr kinases is roughly 10 times higher than the number of Ser/Thr phosphatases [15]. However, the number of protein tyrosine kinases (PTKs) and protein tyrosine phosphatases (PTPs) is comparable [16]. All PTPs share a conserved 240-residue catalytic domain, where there is an invariable signature of 21 residues [17], with a conserved cysteine that is required for catalysis [18]. PTP activity can be regulated through reversible oxidation [19], because at physiological pH the conserved cysteine residue at the catalytic site is in the form of a thiolate anion due to its low pKa (6.0), making it susceptible to oxidation [20]. Oxidation to sulfenic acid can be reversible, while oxidation to the sulfonic acid form is irreversible. 

PTPs oppose to the action of PTKs, so PTP inactivation by reversible oxidation would contribute to sustain the activation of signaling pathways driven by tyrosine phosphorylation. Reduction and reactivation of PTPs by glutathione [21] or thioredoxin [22] would be required for turning off the signaling pathway. This notion is supported by experimental evidence showing the inactivation of different PTPs in response to extracellular signals [23,24]. Serine-threonine phosphatases can also be regulated by oxidation [25].

Bearing the foregoing in mind, it seems that ROS production, through phosphatases oxidation and inactivation, would be linked to the activation of signaling cascades driven by protein phosphorylation. However, it is necessary to point out that kinases are not immune from being regulated by oxidation [26]. Oxidation can lead to the activation of some kinases, such the Src kinase. Although, in contrast, there are also examples in which kinase oxidation can lead to inactivation, such as mitogen-activated protein kinase kinase kinase 1 (MEKK1), protein kinase A (PKA) and protein kinase B (PKB or AKT) [27].

Intracellular calcium levels are important for the regulation of signaling pathways. There is an interesting cross-talk between ROS and Ca^2+^ levels. As recently reviewed [28], ROS can regulate the activity of calcium channels located at the plasma membrane, the mitochondria or the reticulum. For instance, the sarco/endoplasmic reticulum Ca^2+^ ATPase (SERCA) 2b can be modified by *S*-glutathionylation, which enhances its activity [29], leading to a Ca^2+^ influx in the cytoplasm. There is experimental evidence showing that SERCA glutathionylation depends on ROS production by the NADPH oxidases Nox2 and Nox4 [30]. On the other hand, an increase of Ca^2+^ concentration can induce ROS production through the mitochondria [28] or through NADPH oxidases [31]. 

It is well known that gene expression can be affected by intracellular ROS levels. In order to combat oxidative stress, the cell upregulates the expression of genes encoding antioxidant and detoxifying enzymes. These genes are under the control of cis-activating sequences (antioxidant response elements or ARE) that can be bound by Nrf1 and Nrf2 (nuclear factor erythroid-derived 2-related factors 1 and 2) [32].

All signaling cascades terminate in the nucleus, regulating gene expression. Thus, redox signaling can affect gene expression through the regulation of signaling proteins in the cytoplasm as mentioned above. Moreover, there are also a number of transcription factors whose functions can be regulated by reversible oxidation. The oxidation of p53 alters its conformation, inhibiting DNA-binding [33]. The DNA-binding of HLF (HIF-1α-like factor) depends on the reducing conditions. HLF contains one cysteine residue in its DNA-binding domain that is susceptible to oxidation [34]. The activity of NF-κB can be regulated positively and negatively by ROS. Cysteine oxidation reduces NF-κB ability to bind DNA [35], while the degradation of the NF-κB inhibitor IκB can also be triggered by an increase in ROS [36], which would lead to NF-κB activation. Other examples of transcription factors regulated by reversible oxidation are Pax5 [37], Pax8 [38], early growth response-1 (Egr-1) [39], and activator protein-1 (AP-1) [40].

The complex APE1/Ref-1 (AP endonuclease 1/redox factor 1), which is involved in base excision DNA repair, can also regulate the redox state of different transcription factors, promoting their reduction and an increase of DNA-binding [41].

The epigenetic control of transcription is of paramount importance in eukaryotes. Some proteins involved in this control might also be regulated by redox mechanisms. For example, cysteine oxidation can inhibit the catalytic activity of some HDACs (histone deacetylases) [42].

Finally, DNA can also be altered by oxidation. Guanine oxidation generates 8-hydroxyl-2ʹ-deoxyguanosine, which can inhibit DNMTs (DNA-methyl transferases), rendering a local DNA demethylation [43,44]. In addition, 5-methylcytosine oxidation could also lead to demethylation of CpG islands [45], while DNA oxidation prevents the binding of MBPs (methyl-CpG-binding proteins) [46].

In summary, ROS can affect cellular signaling and gene expression through the regulation of certain proteins directly involved in these processes. However, if we consider ROS as bona fide second messengers, we should be able to understand how they reach specificity. One way of reaching such specificity would be the compartmentalization of ROS production. It is also possible that the sensitivity to oxidation may be different among the signaling proteins affected by ROS. 

## 2. The NADPH Oxidase Family

There are several enzymatic systems capable of ROS production in eukaryotes, including the mitochondria, xanthine oxidase, the cytochrome p450 mono-oxygenase, uncoupled NO synthase (NOS), myeloperoxidase, cyclo-oxygenase, and the NADPH oxidase family (Nox). All of these sources, except the Nox family, produce ROS as a collateral effect of their main activity. The Nox family is the only cellular enzymatic system whose principal function is the production of ROS. Moreover, after the mitochondria [47], they seem to be the second most important ROS producer in quantitative terms [48]. The fact that these enzymes are specialized in ROS production, very often in response to extracellular signals, strongly suggests that they could be central players of the so-called redox signaling.

NADPH oxidases are multiprotein complexes associated with membranes. The first identified member of the family was the phagocyte oxidase, required for killing pathogens through the respiratory burst [49]. Genetic defects related to this oxidase complex produce the chronic granulomatous disease (CGD) found in humans [50]. Surprisingly, non-phagocytic cells from CGD patients exhibit normal levels of superoxide, which led to the search for other structurally different NADPH oxidases. Thus, Nox2 (also known as gp91^phox^), the catalytic subunit of the phagocyte oxidase, is the founding member of the family, to which six other members belong (Nox1, Nox3, Nox4, Nox5, Duox1, and Duox2). All of these proteins are transmembrane flavoproteins, showing six transmembrane-spanning α-helix, NADPH- and Flavin adenine dinucleotide (FAD)-binding sites, and four conserved haem-binding histidines at the third and fifth transmembrane helices [49]. 

The stability of Nox2 depends on tight association with the small transmembrane protein p22^phox^, the dimer also known as cytochrome b558 [51]. In phagocytes, this dimer can be found at the plasma membrane or in intracellular vesicles [52]. The formation of the active oxidase complex depends on the assembly of several cytosolic proteins—p47^phox^, p67^phox^, p40^phox^, and the GTPase Rac1—which help to organize the complex [49]. It seems that Nox2 could produce ROS at both locations: at the plasma membrane [52] and in intracellular vesicles [53]. Although Nox2 was originally described in phagocytes, it is present in a wide number of tissues and cell types [49], including endothelial cells [54].

The Nox1 protein shows a 56% protein sequence identity with Nox2 [55]. Nox1 is highly expressed in colon epithelium, but is also present in other tissues such as the prostate, uterus, and vascular smooth muscle [56], and in endothelial cells [57]. Moreover, Nox1 expression is inducible under different stimuli, such as angiotensin II, platelet-derived growth factor (PDGF) [58], and prostaglandins [59]. Like Nox2, Nox1 is also associated with p22^phox^ [49] and requires other cytosolic subunits for its activity, such as NOXO1 (NOX organizer 1, a homologue of p47^phox^), NOXA1 (NOX activator 1, a homologue of p67^phox^), and the Rac GTPase [60].

Nox3 shows 58% identity with Nox2 at the protein level [61] and is highly expressed in the inner ear. Its deficiency can cause equilibrium problems, as illustrated by the “head tilt” phenotype observed in mice with *Nox3* gene mutations [62]. Nox3 can be also found at low levels in other tissues [61], mainly in fetal tissues such as fetal spleen, kidney, and skull, suggesting that it could be the most important NADPH oxidase for tissue development. Nox3 is also dependent on p22^phox^ protein [63]. In fact, there are p22^phox^ point mutations that also lead to the “head tilt” phenotype [64]. Nox3 activity also seems to depend on NOXO1 [61,65], but not on NOXA1 [66].

Nox4 is a more distant homologue of Nox2 than Nox1 and Nox3. The identity between Nox4 and Nox2, at the protein level, is 39%. Nox4 shows a wide tissue distribution, including endothelial cells [67], with a high expression in the kidney [68]. Nox4 is also stabilized by p22^phox^ [69], but its activity does not require a cytosolic subunit. It is suggested that Nox4 might be constitutively active, producing H_2_O_2_ rather than superoxide [70]. However, it can also be activated by several extracellular stimuli such as phorbol esters [71], insulin [72], and angiotensin II [73]. 

Nox5 has been found to be expressed in human spleen, testis, lymphoid tissues, and endothelial cells [74] and has also been found to be highly expressed in several cancer cell lines [75,76,77]. In contrast to Nox1, Nox2, Nox3, and Nox4, Nox5 is p22^phox^-independent and contains an EF-hand domain, indicating that Nox5 activity can be regulated by calcium [78]. Furthermore, there is also a shorter version of the protein that lacks the EF-hand motif, called Nox5-S [79].

Duox1 and Duox2 are highly expressed in the thyroid [80,81] and share a 50% identity with Nox2. Their structures are very similar to those of other Nox proteins, although Duox1 and -2 present a seventh transmembrane helix that protrudes out of the membrane in a peroxidase-like domain. Nevertheless, it is unclear whether this domain has catalytic activity [49]. Like Nox5, Duox1 and 2 possess an EF-hand region [82] and can therefore be activated by Ca^2+^. In contrast to Nox5, Duox1 and -2 require the presence of subunits DuoxA1 or DuoxA2 for their activity [83].

The different members of the NADPH oxidase family show a particular pattern of expression. In addition, it is intriguing that most of the cells express different Nox isoforms. Bearing this in mind, it could be suggested that there is a specificity of function among the different Nox isoforms, both at the tissue and cellular level. One way of achieving such specificity, within the same cell, could be through different cellular locations. The main subcellular location for most NADPH oxidases is the plasma membrane [64,74,84,85]; although they can also be found in other intracellular membranes. Nox1 is specifically located in caveolae regions, and in intracellular endosomes [84,85], and Nox1, Nox2, Nox4, and Nox5 have been localized in perinuclear areas [74,86]. It seems that Nox2 colocalizes with the nuclear pore complex [87]. Nox4 seems to be mainly localized at the reticulum [88], but also appears at the mitochondria [89], at focal adhesion [88], and even in the nucleus [71]. Duox1 and Duox2 appear to be initially located in the endoplasmic reticulum, and, in the presence of their maturation factors, DuoxA1 and DuoxA2, are transferred to the plasma membrane [83].

## 3. Redox Signaling in Angiogenesis

Endothelial cells (ECs) occupy the inner surface of blood vessels and not only act as a barrier between the blood and other tissues but also adopt an active role in the control of vascular tone, permeability, thrombogenesis, inflammation, and immunity [90]. Upon an increase of shear stress, ECs produce vasodilators, such as nitric oxide (NO) or prostacyclin [91], highlighting the importance of ECs in the control of vascular tone and blood pressure.

Resting ECs express and release anti-coagulant and anti-adhesive molecules, which prevent platelet aggregation and blood coagulation [92]. However, upon vascular injury, ECs are activated and produce pro-thrombotic mediators such as Von Willebrand factor [93]. In addition, ECs, by increasing the expression of cell adhesion molecules such as vascular cell adhesion molecule 1 (VCAM-1) [94] and intercellular adhesion molecule 1 (ICAM-1) [95], enhance platelet and leukocyte adhesion and aggregation [90]. Moreover, the breakage of the endothelium barrier allows access to pro-adhesive and pro-thrombotic macromolecules from the subendothelium, leading to platelet activation and eventually to blood coagulation [96].

In addition to the aforementioned, when required, ECs initiate angiogenesis, the process of the formation of new blood vessels from pre-existing vessels. Redox signaling is important for ECs function, and it has been shown that it can trigger angiogenesis [97,98,99,100]. The importance of redox signaling for angiogenesis was described by numerous reports showing that H_2_O_2_ can trigger this phenomenon [101,102]. In fact, in heart ischemia [103,104] or anaesthesia [105], an increase in ROS production that activates neovascularization has been observed. On the contrary, antioxidant treatment has an inhibitory effect on angiogenesis [106,107].

Angiogenesis requires ECs migration and proliferation, but in order for this to occur, quiescent ECs must first become detached from the basal membrane. These three different stages are activated in response to angiopoietin and vascular endothelial growth factor (VEGF) [108,109,110], and ROS production seems to be important throughout the entire process [111]. During the detachment of ECs from the basal membrane, ROS production facilitates the phosphorylation of VE-cadherin and β-catenin, allowing the dismantling of the vascular endothelial-cadherin (VE-cadherin) tight junctions [112]. Localized ROS production is also observed at the membrane ruffles of migrating ECs [113,114,115]. Finally, proliferating ECs show higher levels of ROS than do quiescent ECs [116], which appears to be important for the activation of proliferating signaling pathways [117,118].

**Figure 1:**
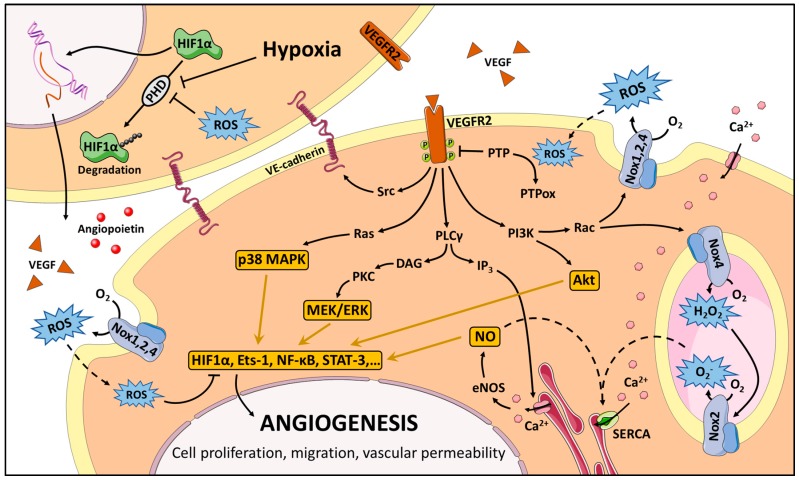
Role of ROS in VEGF-mediated angiogenesis. Stabilization of HIF1α by hypoxia or by an increase of ROS leads to the expression of several proangiogenic factors such as VEGF and angiopoietin. VEGF stimulates multiple signaling pathways in ECs, with a simultaneous ROS production by NADPH oxidases. ROS would help to maintain the VEGF signaling pathway activated through the inhibition of PTPs. Some Ca^2+^ channels can be regulated by ROS. Finally, ROS can modulate the activity of transcription factors involved in the regulation of angiogenesis. More details in the text. (Figure created from the image bank of Servir Medical Art, licensed under Creative Commons).

We are beginning to understand the underlying mechanism regarding how ROS regulates angiogenesis (Figure 1). In order to fully comprehend how this system works, we need to first know how ROS production is regulated, and then identify ROS molecular targets. NADPH oxidases-driven ROS production in ECs can be activated by several extracellular messengers (VEGF, transforming growth factor β or TGFβ, and angiotensin II), or by shear stress. Consequently, ROS can behave as second messengers, contributing to the signaling that leads to EC migration and proliferation [119]. 

ROS production by NADPH oxidases is required for the regulation of the vascular system. Several Nox isoforms are expressed in arteries and endothelial cells (Nox1, Nox2, Nox4, and Nox5) [120], while only Nox2 and Nox4 appear to be expressed in their progenitors cells [121]. It seems that, in veins, Nox2 is the predominant isoform [122]; however, Nox4 is the most abundant isoform in endothelial cells [67,123]. As discussed above, Nox subcellular location [124] must also be considered for understanding the relevance of this family of enzymes for EC function. It has been described that Nox1, Nox2, and Nox4 can be simultaneously expressed in a given EC. The three Nox proteins appear in perinuclear regions, and Nox2 and Nox4 are also present at the endoplasmic reticulum, while Nox2 is present at the plasma membrane [86].

The importance of Nox function for cardiovascular physiology has been well illustrated by the use of knock-out animal models and cell-specific studies. Nox1-deficient mice show a moderate decrease of basal blood pressure and do not respond to angiotensin II [125]. ROS production in response to angiotensin II is related to hypertension, and experimental evidence suggests that Nox1 ROS production is required for angiotensin II hypertension [126].

The induction of Nox2 activity in ECs by angiotensin II shows a strong profibrotic effect, eventually leading to diastolic cardiac function and diastolic hypertension [127]. Specific deletion of Nox2 in myeloid cells reduces blood pressure, suggesting that, while endothelial Nox2 regulates angiotensin II hypertension, myeloid Nox2 is involved in the regulation of basal blood pressure [128].

Nox5 expression and activity is increased during hypertension and atherosclerosis, suggesting the importance of Nox5 ROS production for vascular remodeling in hypertension [129].

According to in vitro results, Nox1, Nox2, and Nox4 can activate ECs proliferation and tubulogenesis. In vivo studies also indicate that these three Nox isoforms are implicated in angiogenesis [130]. However, the relative importance of the different Nox isoforms in angiogenesis is still under debate. Nox5 is not present in rodents, and as a result the study of their function in angiogenesis lags behind due to the lack of suitable animal models; however, an increase of Nox5 expression and activity in coronary artery disease [131] and in atherogenesis [132] has been reported. 

ROS production by Nox1 is required for smooth muscle cell migration [133]. Inhibition of Nox1 seems to impair EC migration and tubulogenesis in vitro [57,134]. In vivo models show that the deficiency of Nox1 in mice, but not Nox2 and Nox4, impaired angiogenesis in bFGF-loaded matrigel implants [135].

Several reports suggest the importance of Nox2 for ECs proliferation [86,136,137], and in vivo studies have shown the importance of Nox2 for angiogenesis. Tojo et al. demonstrated that, in a mouse ischemic hindlimb model, neovascularization is seriously compromised by Nox2 deficiency. In the wild-type control animals, an increase in ROS and inflammation, together with increased Nox2 expression, were detected three days after injury. Seven days after injury, neovascularization was noticeable, and Nox2 expression and ROS levels remained high. The use of an ROS scavenger allowed these authors to suggest that increased ROS production is required for neovascularization. Moreover, the fact that the formation of new vessels is compromised in Nox2-deficient animals strongly suggests the relevance of Nox2-dependent ROS production for in vivo angiogenesis in this animal model [138]. Neovascularization upon ischemia requires angiogenesis and vasculogenesis, the formation of new vessels from the mobilization of endothelial progenitor cells (EPCs) from bone marrow. Interestingly, it has been shown that upon ischemia, Nox2 expression is activated in bone marrow mononuclear cells, together with an increase of circulating EPCs. In vivo Nox2 deficiency impairs EPCs mobilization and neovascularization [139], suggesting the importance of Nox2 ROS production for EPCs mobilization and homing, an event that is required for neovascularization in response to ischemia. In addition to its implication in ischemia-induced angiogenesis, Nox2 also seems to be required for in vivo angiogenesis in response to VEGF [140] and urotensin II [141]. This latter study showed how urotensin II promotes Nox2-dependent ROS production, leading to the stabilization of HIF1α, which then activates the expression of Nox2 gene, leading to a further increase in ROS production required for angiogenesis.

Although the aforementioned studies are in agreement with the notion that Nox2 ROS production is required for angiogenesis, it is important to highlight that some studies have shown how Nox2 overactivation is detrimental for angiogenesis. It has been reported that in diabetic animals there is an exacerbated ROS production associated with an increased expression of Nox2 [142]. This study showed how neovascularization after ischemia is impaired in diabetic animals, which could be rescued by blocking Nox activity. Moreover, hypercholesterolemia is another pathology in which impaired neovascularization upon ischemia has been related to oxidative stress. Haddad et al. revealed how hypercholesterolemia induces Nox2 expression in ischemic tissues of wild-type animals, which is associated with an impaired neovascularization [143]. In contrast, under the same conditions, improved neovascularization was observed in Nox2-deficient animals. Thus, it could be argued that Nox2-dependent redox signaling would be required for angiogenesis, while oxidative stress appears to be detrimental for the same process.

Nox4 contributes to ROS production in both smooth muscle cells [144] and ECs [67]. In vitro studies show how Nox4 activates angiogenic responses in cultured human ECs [145], and more recently it has been shown that Nox4 is required for proliferation and migration of human EPCs [146], suggesting the importance of Nox4 for neovascularization. Different in vivo models suggest the relevance of Nox4 for angiogenesis. The expression of Nox4 is activated under hypoxia, which seems to be required for angiogenesis [117]. This study showed that overexpression of Nox4 in ECs enhanced recovery after surgery in the hindlimb ischemia model. The authors suggest that Nox4 induced angiogenesis by enhancing endothelial nitric oxide synthase (eNOS) expression and activity. These results have been corroborated by recent studies in which, in addition to eNOS, the importance of heme oxygenase-1 (HO-1) [118], TGFβ signalling, and VEGFR2 expression [147] for Nox4 proangiogenic effect has been demonstrated.

The group led by Shah demonstrated that, while Nox2 mediates cardiac hypertrophy upon pressure overload [148], Nox4 showed a protective effect [149]. In a later study, these authors showed how Nox4 expression in cardiomyocytes is enhanced by pressure overload. Under this type of stress, a Nox4 knockout model was observed to be more sensitive than wild-type animals, showing an exacerbated cardiac hypertrophy and fibrosis. Transgenic mice overexpressing Nox4 in cardiomyocytes were protected against pressure overload. These mice showed more myocardial capillarity than the control animals, suggesting that overexpression of Nox4 upon cardiac stress is important for the protection against cardiac dysfunction. This study showed that Nox4 proangiogenic effect is mediated by the increase of HIF1α and VEGF levels.

Recently, it has been shown that Nox4 is required for exercise-induced angiogenesis in skeletal muscle [150], and for tumor angiogenesis [151]. Therefore, all of these findings are in agreement with the pivotal role that Nox4 plays in angiogenesis.

Although we have focused on the importance of NADPH oxidase family for angiogenesis, it is worth bearing in mind that other sources of ROS, such as mitochondria and xanthine oxidase, can also be involved in angiogenesis [97]. Moreover, we must not overlook the fact that redox signaling depends on the balance between ROS production and elimination. Therefore, the implication of cellular antioxidant defences in the regulation of angiogenesis would be required. Glutathione (GSH) is one of the main cellular antioxidant defences. Additionally, there are reports suggesting that an increase of GSH would hamper angiogenesis. The molecular mechanisms driving this effect are yet not understood, but the effect could, at least in part, be related to the regulation of HIF1α activity that would decrease upon high GSH levels [97]. In contrast, the expression of heme oxygenase-1 (HO-1), an enzyme that intervenes in heme degradation and protects the cells against oxidative stress, shows proangiogenic effects [152]. Future studies are required for a better understanding of the implication of antioxidant defences in angiogenesis.

The family of VEGF glycoproteins are central regulators of angiogenesis. H_2_O_2_ treatment can induce the expression of VEGF [153] and VEGF receptor 2 (VEGFR2) [154]. This could somehow explain why H_2_O_2_ treatment has a proangiogenic effect. In ECs, VEGF can bind to two different receptors with intrinsic tyrosine kinase activity (RTKs), VEGF receptor-1 (VEGFR1 or Flt-1) and VEGFR2 (also known as Flk1/KDR). Activation of VEGFR2 promotes ECs proliferation and migration [155]. Like other RTKs, VEGFR2 activation begins by receptor dimerization and transphosphorylation, and then several signaling cascades are activated downstream, such as mitogen-activated protein kinases (MAPKs), PI3k/AKT, or eNOS [156].

It has been shown that VEGF induces ROS formation through Nox2 and Nox4 [157]. ROS production seems to be required for VEGF receptor phosphorylation [140]. ROS could facilitate signal transduction through several mechanisms. The proangiogenic effect of PTP chemical inhibitors [158] suggests that the inhibition of phosphatases might be important for angiogenesis. There are a number of phosphatases that can have an impact on VEGFR2 signaling, recently reviewed by Corti and Simons [159]. ROS-dependent inactivation of some of these phosphatases would facilitate VEGF signal transduction. There are already some examples supporting this working model, such as the inhibition of SHP1 [160], LMW-PTP [161], PTP1B, and DEP1 [162].

The cross-talk between ROS and Ca^2+^ homeostasis might be important during angiogenesis. It has been shown that, in response to nitric oxide, ROS can induce the activation of SERCA Ca^2+^ channels by glutathiolation [29]. The activation of Nox4 ROS production in response to VEGF seems to be required. Nox2 and eNOS would participate in such activation downstream of Nox4. Eventually this determines the entry of Ca^2+^ into the endoplasmic reticulum through SERCA Ca^2+^ channels and into the cytoplasm through plasma membrane-associated channels. Such Ca^2+^ influx is required for ECs migration [30].

ROS can influence VEGF signaling and angiogenesis through the regulation of transcription. It has long been known that exogenous H_2_O_2_ can stimulate the expression or the activity of transcription factors required for angiogenesis, such as Ets-1 [102], NF-kB [163], and STAT3 [164]. Moreover, there are a number of important genes required for angiogenesis whose expression is ROS-dependent, such as monocyte chemoattractant protein-1 (MCP-1) [165], vascular cell adhesion molecule 1 (VCAM-1) [166], and matrix metalloproteinases (MMPs) [167].

The transcription factor HIF1α displays a prominent position in the regulation of hypoxia-induced angiogenesis. The stabilization of HIF1α under hypoxia drives the expression of VEGF [168], angiopoietin [169], erythropoietin [170,171], and SDF-1 [172]. All of these cytokines have angiogenic properties [130]. There are two different phases of HIF1α hypoxic stabilization. In the early stage, HIF1α stabilization depends on mitochondrial ROS production [173], and on VEGF production [174]; then, VEGF stimulates the production of ROS through NADPH oxidases [175], which reinforces HIF1α stabilization. Interestingly, HIF1α also controls Nox4 promoter activity [176].

Redox signaling termination during angiogenesis can be facilitated through ROS scavenging by cellular antioxidant systems [177]. Moreover, it is important to note that VEGFR2 can be directly modified by ROS, through the formation of an inactivating intramolecular disulphide bridge [178]. This could be a feed-forward negative regulation system important for signal termination (see Figure 1).

## 4. Therapeutic Opportunities

EC homeostasis can be altered by oxidative stress, leading to vascular-related diseases [179], such as atherosclerosis [180], hypertension [181,182], cancer [100], and diabetic retinopathy [183]. Among the different sources of ROS linked to vascular diseases, NADPH oxidases occupy a prominent position. Under physiologic conditions, the production of ROS by vascular Nox is supposed to be low; however, overactivation of these enzymes can lead to pathological conditions [184].

One of the first diseases related to NADPH oxidases was hypertension [185]. It seems that the production of ROS by Nox functions as a hypertensive signal [186,187]. The difficulty lies in delineating the importance of the different Nox isoforms for hypertension. Angiotensin II is strongly related to the development of this disease, and increased NADPH oxidase activity seems to be required for angiotensin II induced hypertension [185]. Nox1 and Nox2 deficiencies in mice hamper the hypertensive response to angiotensin II [125,126,188], while Nox1 [189] and Nox2 [190] overexpression increases the hypertensive response to angiotensin. All of these findings suggest that the production of ROS by Nox1 and Nox2 is associated with hypertension. Overexpression of Nox4 in the ECs of transgenic mice leads to vasodilation and decreased blood pressure [191], suggesting that Nox4 derived ROS could have a protective effect against hypertension. However, a later study did not find any effect on the angiotensin II hypertensive response in *Nox4*-deficient mice [118]. A recent report suggests that Nox4 activity could be required for hypertension mediated by chronic intermittent hypoxia, which could be related to the Nox4 increased expression in the kidney [192]. Interestingly, there are in vivo studies showing an implication of renal Nox4 in the development of salt-induced [193] or angiotensin-induced hypertension [194]. Nox4 has been also related to the development of pulmonary hypertension [195]. Therefore, the relation of Nox4 with hypertension is complex; however, it is tempting to speculate that, depending on its tissue distribution, Nox4 could behave as a protector or inductor of hypertension. 

Much effort has been invested in studying the implication of the NADPH oxidase family in the development of atherosclerosis [184]. In humans, genetic disorders affecting Nox2 phagocyte oxidase lead to the occurrence of chronic granulomatous disease (CGD). This ailment is associated with a lower risk of atherosclerosis [196]. Nox1 [197] and Nox4 [198] are overexpressed in atherogenic regions. These findings, among others, [131,199], would support the hypothesis that ROS production by the NADPH oxidase family would contribute to the development of atherosclerosis. However, experiments in animal models offer conflicting results: some reports suggest that Nox2 [200] or *p47^phox^* [201] deficiencies do not affect the development of atherosclerosis in *ApoE^−/−^* mice, while others suggest that the lack of *p47^phox^* reduces the atherosclerotic lesion progression [202]. The differential expression of the NADPH family in the vascular system [198] makes the study of the implication of this family in atherosclerosis difficult. However, there seems to be a consensus in the field that NADPH oxidase targeting would be a good strategy against this disease.

Vascular problems are one of the most threatening complications for patients with diabetes. Some reports suggest the involvement of Nox1 and Nox4 in diabetic vascular ethology [203]. A common complication is the development of diabetic retinopathy (DR) [204]. Oxidative stress has been related with the onset of DR, and NADPH oxidase seem to be one of the most important ROS sources, which lead to this pathological condition [130].

Tumor cells express higher levels of ROS than normal cells [5]. Such exacerbated ROS production could induce angiogenesis during tumor progression [101], while antioxidants may have the opposite effect [205]. Experiments in animal models point to the importance of Nox1 [135] and Nox2 [139,140] for tumor neovascularization. Therapeutic strategies modifying ROS levels in cancer cells have drawn attention in the past few years. The experimental evidence is consistent with the notion that increasing ROS levels would be detrimental for tumor cells, because they would reach an unbearable state of oxidative stress. Under the same conditions, healthy cells would be spared from crossing this threshold. Therefore, the search of pro-oxidants treatments against cancer over the last few years has been very proactive [5,206]. On the other hand, given that ROS fuels cancer cell proliferation it could be surmised that avoiding ROS production and ROS scavenging could also be a suitable anti-tumor strategy [5]. Cancer progression depends on blood vessel formation; therefore, hampering angiogenesis is regarded as a suitable anti-tumor strategy. As discussed earlier, an excessive ROS production could hamper angiogenesis, so the pro-oxidant strategy would not only kill tumor cells, but would also reduce the blood supply, while, conversely, ROS scavenging would prevent or mitigate tumor angiogenesis [100].

Following the same reasoning, ROS scavenging could be also applicable to the treatment of vascular diseases, given the implication of oxidative stress in these diseases. It is commonly believed that the use of antioxidants is beneficial for human health, and for reducing cellular oxidative stress. It could be presumed that antioxidants intake could prevent cardiovascular diseases or cancer. However, this belief is not supported by solid scientific evidence [207]. Given the importance of ROS for the control of cellular signaling and gene expression, the use of an ROS-scavenging strategy is perhaps a very rough approximation that could negatively affect important cellular processes. Although altering ROS levels may be therapeutically beneficial, we should still try to provide more specific strategies than just the use of antioxidants, such as targeting cellular ROS producer systems. In this case, targeting NADPH oxidases seems an appealing strategy against cardiovascular diseases [208]. However, there are some difficulties that need to be overcome in the future in order to achieve effective treatments. First, we still do not know the precise role of each Nox isoform in physiologic and pathologic angiogenesis. Therefore, in order to design effective treatments, we would need further investigation to delimitate the most convenient target for each pathological condition is needed. Second, the field desperately needs the development of novel, more specific and selective, Nox inhibitors. Historically, diphenylene iodonium (DPI) and apocynin have been used to inhibit NADPH oxidase activity; however, they are neither specific nor selective. DPI is a flavoprotein inhibitor, while apocynin blocks NADPH oxidase assembly, although it seems to be non-effective against vascular Nox [209]. A great effort is currently being made to find and develop new inhibitors. Ideally these new inhibitors should not affect other flavoproteins and should not behave as antioxidants. As recently reviewed [208,210], there is a number of newly discovered small molecules that can inhibit NADPH oxidase activity (Table I). GKT136901 and GKT137831 strongly inhibit Nox1, Nox4, and Nox5 and are less effective against Nox2. These two inhibitors are quite promising, because they are orally bio-available and GKT137831 is under clinical development. Ebselen and its derivatives have shown strong inhibiting activity against several Nox. Ebselen is also orally available, but it has also been shown to have a potent peroxynitrite scavenger activity in vitro.

Some natural derivatives show Nox-inhibiting activity; celastrol, derived from Thunder God Vine, inhibits Nox1, Nox2, and to a lesser extent Nox4 and Nox5. However, this compound has other targets such topoisomerase II or proteasome. The natural environment could be a good source of novel Nox inhibitors, as shown by the wide range of compounds that have been described to have such effects, which include diarylheptanoids, grindelic acic, procyanidincs, flavonoids, Britannica L, flavonoids, and ginkgolide B.

Since it has proven difficult to find small molecules specifically targeting Nox isoforms, another line of research could involve the development of Nox-specific inhibitors. Two peptides inhibitors have been designed for the specific inhibition of Nox2 (Nox2dstat) and Nox1 (Noxa1ds) by blocking the assembly of the Nox complex. 

## 5. Conclusions

The importance of NADPH oxidase ROS production plays a central role in the control of cellular signaling in eukaryotic cells. There is a plethora of reports showing the importance of redox signaling for angiogenesis, both in physiologic and pathologic conditions. Over the last two decades, much effort has been made to decipher the role of different Nox isoforms involved in this process. A better understanding of redox signaling during angiogenesis will allow the design of novel therapeutic strategies, based on targeting the Nox family alone or in combination with other important components of this process.

## Figures and Tables

**Table I:** Selectivity of some NADPH oxidase inhibitors.

Inhibitor	Main Target	Other Targets	Side Effects	Reports in Angiogenesis
GKT136901	Nox1, Nox4 and Nox5	Nox2	Peroxynitrite scavenger	Inhibition of tumor angiogenesis in mouse models [135]
GKT137831	Nox1, Nox4 and Nox5	Nox2	None tested	
ML171	Nox1	Nox2, Nox3, Nox4 and XO	Inhibitor of serotonin and adrenergic receptors	Inhibition of vasculogenesis in embryoid bodies [211]
VAS2870	Nox2	Nox4 and Nox5	Thioalkylate RyR1 and GSH	Inhibition of vasculogenesis in embryoid bodies [211]
VAS3947	Nox1, Nox2 and Nox4	Nox4 and Nox5		
S17834	Nox *		AMPK activation	
Fulvene-5	Nox2 and Nox4	None tested	None tested	
Triphenylmethane derivates	Nox2 and Nox4	None tested	None tested	
Ebselen	Nox1, Nox2 and Nox5		Peroxynitrite scavenger in vitro, and eNOS inhibitor	Inhibits in vitro ECs migration induced by SCDF-1a [212]
Celastrol	Nox1 and Nox2	Nox4 and Nox5	Inhibitor of topoisomerase II and proteasome	Inhibition of tumor VEGF-induced BM-EPC- supported vasculogenesis in vitro [213] Inhibition of macrophage-induced corneal neovascularization in rats [214]

Abbreviations: XO: xanthine oxidase; RyR1: ryanodine receptor Ca^2+^ channel; GSH: reduced glutathione; AMPK: adenosine monophosphate-activated protein kinase; eNOS: endothelial NO synthase; SCDF-1a: Stromal Cell-Derived Factor-1α; * = Selectivity not tested.
